# A point-of-care clinical trial comparing insulin administered using a sliding scale *versus* a weight-based regimen

**DOI:** 10.1177/1740774511398368

**Published:** 2011-04

**Authors:** Louis D Fiore, Mary Brophy, Ryan E Ferguson, Leonard D’Avolio, John A Hermos, Robert A Lew, Gheorghe Doros, Chester H Conrad, Joseph A (“Gus”) O’Neil, Thomas P Sabin, James Kaufman, Stephen L Swartz, Elizabeth Lawler, Matthew H Liang, J Michael Gaziano, Philip W Lavori

**Affiliations:** a Massachusetts Veterans Epidemiology Research and Information Center (MAVERIC), VA Cooperative Studies Program, VA Boston Healthcare System, Boston, MA, USA; b Department of Epidemiology, Boston University School of Public Health, Boston, MA, USA; c Department of Medicine, Boston University School of Medicine, Boston, MA, USA; d Center for Surgery and Public Health at Brigham and Women’s Hospital, Harvard Medical School, Boston, MA, USA; e Section of General Internal Medicine, Boston Medical Center, Boston, MA, USA; f Department of Biostatistics, Boston University School of Public Health, Boston, MA, USA; g Department of Medicine, Brigham and Women’s Hospital, Boston, MA, USA; h Department of Medicine, Harvard Medical School, Boston, MA, USA; i Division of Rheumatology, Immunology, and Allergy, Brigham and Women’s Hospital, Boston, MA, USA; j Health Research and Policy, Stanford University, Palo Alto, CA, USA; k Department of Statistics, Stanford University, Palo Alto, CA, USA

## Abstract

***Background*** Clinical trials are widely considered the gold standard in comparative effectiveness research (CER) but the high cost and complexity of traditional trials and concerns about generalizability to broad patient populations and general clinical practice limit their appeal. Unsuccessful implementation of CER results limits the value of even the highest quality trials. Planning for a trial comparing two standard strategies of insulin administration for hospitalized patients led us to develop a new method for a clinical trial designed to be embedded directly into the clinical care setting thereby lowering the cost, increasing the pragmatic nature of the overall trial, strengthening implementation, and creating an integrated environment of research-based care.

***Purpose*** We describe a novel randomized clinical trial that uses the informatics and statistics infrastructure of the Veterans Affairs Healthcare System (VA) to illustrate one key component (called the point-of-care clinical trial – POC-CT) of a ‘learning healthcare system,’ and settles a clinical question of interest to the VA.

***Methods*** This study is an open-label, randomized trial comparing sliding scale regular insulin to a weight-based regimen for control of hyperglycemia, using the primary outcome length of stay, in non-ICU inpatients within the northeast region of the VA. All non-ICU patients who require in-hospital insulin therapy are eligible for the trial, and the VA’s automated systems will be used to assess eligibility and present the possibility of randomization to the clinician at the point of care. Clinicians will indicate their approval for informed consent to be obtained by study staff. Adaptive randomization will assign up to 3000 patients, preferentially to the currently ‘winning’ strategy, and all care will proceed according to usual practices. Based on a Bayesian stopping rule, the study has acceptable frequentist operating characteristics (Type I error 6%, power 86%) against a 12% reduction of median length of stay from 5 to 4.4 days. The adaptive stopping rule promotes implementation of a successful treatment strategy.

***Limitations*** Despite clinical equipoise, individual healthcare providers may have strong treatment preferences that jeopardize the success and implementation of the trial design, leading to low rates of randomization. Unblinded treatment assignment may bias results. In addition, generalization of clinical results to other healthcare systems may be limited by differences in patient population. Generalizability of the POC-CT method depends on the level of informatics and statistics infrastructure available to a healthcare system.

***Conclusions*** The methods proposed will demonstrate outcome-based evaluation of control of hyperglycemia in hospitalized veterans. By institutionalizing a process of statistically sound and efficient learning, and by integrating that learning with automatic implementation of best practice, the participating VA Healthcare Systems will accelerate improvements in the effectiveness of care.

## Introduction

Medical decision making is informed by clinical trials and observational studies. Randomization in clinical trials reduces or eliminates biases of observational studies, such as selection by indication and confounding from unmeasured prognostic factors that affect treatment decisions and outcomes. By their purpose, randomized clinical trials (RCTs) can be designed on a spectrum ranging from *pragmatic* (comparing effectiveness of interventions in the most realistic of situations and with diverse subjects) to *explanatory* (comparing efficacy in precisely described clinical situations and selected patients) [1,2]. The goal of explanatory trials is to better understand how and why an intervention works while pragmatic clinical trials are designed to provide information needed to assist healthcare providers make informed clinical decisions [3].

The *Pragmatic–Explanatory Continuum Indicator Summary (PRECIS)* is a measure of where on this continuum an individual trial is situated [4]. It takes under consideration the attributes of an RCT such as flexibility of the interventions, practitioner expertise required, eligibility criteria, intensity of follow-up and adherence monitoring, and the nature and scope of the primary outcome. RCTs are considered on the pragmatic end of the spectrum when these attributes are chosen to allow the trial to more closely mimic conditions encountered in the clinical care arena. Examples include eligibility criteria that reflect the patient population likely to receive the intervention, study investigators with expertise and experiences similar to the healthcare providers who will ultimately administer the treatments, treatment protocols that allow the flexibility required in routine clinical care, and outcome measures, and follow-up procedures that would be part of routine clinical care. Despite their reflection of routine clinical care, pragmatic trials are currently still complicated and expensive to implement, because of the use of dedicated study personnel to recruit participants, administer the intervention and monitor the participants for study outcomes and adverse events.

We are testing a real implementation of a new methodology for clinical trials, that we have called point-of-care clinical trials (POC-CTs), with features designed to maximize the pragmatic nature of studies. Aspects of the approach we describe here have been proposed or implemented by others [5–8] and discussed in detail under the name of the ‘clinically integrated randomized trial’ by Vickers and Scardino [9]. The defining characteristic here is that to the maximum extent possible the clinical trial apparatus is embedded in routine clinical care. Optimally, this would include recruitment and randomization of study subjects at their POC by their usual healthcare provider. Once randomized to a treatment arm subjects would continue to be treated by their healthcare provider with minimal or no deviation from usual care. Follow-up of participants would thus reflect current clinical practice. Assessment of subject compliance and practitioner adherence to protocol, and ascertainment of clinically relevant endpoints would be performed through medical record review, with minimal contamination of the clinical care ‘ecosystem’ by intrusive study dependencies. The intrusiveness of study operations, from randomization through endpoint ascertainment, would be greatly reduced if performed using tools familiar to healthcare providers and data already present in an electronic medical record (EMR).

A POC-CT shifts away from the asynchronous, distinct, and separate environments of research and clinical care, toward a real-time integrated system of research-based care. The goal of POC-CTs is to deliver the best care to patients while learning from each experience and redefining that care. Under this new paradigm, ongoing results would be more rapidly and more likely adopted by providers who participated in the studies. By synthesizing research with practice and tools to learn from that process, participating facilities can move to the goal of becoming ‘learning healthcare systems.’

In this article, we describe a specific POC-CT designed to test the feasibility and usefulness of the method, in answering a question of relevance to the Veterans Affairs (VA) Healthcare System. The clinical context and issues are described and ethical issues discussed. The use of outcome adaptive randomization to enhance implementation also addresses the frequentist operating characteristics of the design. The kinds of comparativeness questions best suited to POC-CT are argued.

## Illustrative example: sliding scale insulin regimen *versus* weight-based insulin protocol

We describe a POC-CT which compares two common regimens of administering insulin therapy to hospitalized patients requiring insulin; the sliding scale and weight-based approach. The VA has an EMR that includes electronic ordering of medications and protocols for both of these insulin regimens. Review of EMR data at the VA Boston Healthcare System demonstrated that each of these two approaches is used with approximately equal frequency and discussions with treating clinicians indicated that choice of method administration is based on personal preference and not on patient specific determinants.

There are no published data comparing the effectiveness or the adverse effects of the sliding scale or a weight-based insulin protocol in treating inpatients with hyperglycemia. For the sliding scale, short acting insulin is administered three to four times daily according to the degree of hyperglycemia, and no basal insulin is administered. This regimen, therefore, responds to hyperglycemia after it occurs, and does not prevent it. The weight-based insulin protocol is a twice daily regimen of basal intermediate-acting insulin (NPH) plus a pre-meal twice a day regimen of short acting regular insulin, plus a correction dose of regular insulin depending on the degree of hyperglycemia. In addition, depending on the amount of the correction dose, the basal doses are adjusted upward for the next day’s NPH insulin dose to manage the hyperglycemia.

## Study design

Overall, the study is an open-label, randomized trial comparing sliding scale to a weight-based regimen in non-intensive care units (ICU) inpatients in a single large VA healthcare facility. There will be no modification to the treatment protocols already in use which will be accessed through the existing order entry menu. Consented patients will be randomized to treatment arms using an adaptive randomization method. Subjects are otherwise treated as usual. That is to say, there is no treatment protocol imposed other than insulin regimen beyond randomization. *There are no required diagnostic procedures and no study-specific follow-up events required*. Outcomes and covariates data will be collected directly from the computerized patient record system (CPRS). The primary endpoint is hospital length of stay (LOS); secondary endpoints include glycemic control and readmissions for glycemic control within 30 days of hospital discharge. Analysis will be based on intention to treat.

We considered using a cluster-randomized design, but the number of natural clusters (treatment units) within a hospital is small and having enough clusters to achieve adequate power would require opening the study at many hospitals, posing too many complex issues for a first use of POC-CT. Furthermore, we are interested in testing the feasibility of individual patient-level randomization, and the use of adaptive randomization to ‘close the implementation gap.’ While it is possible to imagine an adaptive cluster-randomized design, we have little information on the parameters necessary for design of such a study.

## Eligibility

All non-ICU patients who require sliding scale or weight-based insulin therapy are eligible. The decision to obtain consent from a given individual will be made by the ordering clinician at the time of an insulin order (see section ‘Methods’). There are no exclusions.

## Treatment regimens

The treatment regimens are sliding scale and weight-based insulin as currently operationalized at the VA Boston Healthcare System. The ordering clinician finds these protocols under the electronic endocrine order menu and is led through order entry screens that insure standardization of the treatment protocol. The sliding scale and weight-based insulin regimens order menus in place at the medical center were not modified other than to add a third choice allowing for randomization through the POC-CT mechanism.

## Follow-up

Consenting subjects will be followed until 30 days of post-randomization. Following informed consent subjects will not be contacted by the study team either during their hospitalization or after discharge. All follow-up data will be collected *via* the EMR.

## Data collection

Variables collected include demographics (age and gender); admission date, discharge date, and bed location (acute *vs.* non-acute); bed service (medical, surgical, and other); admission and other medical diagnoses (ICD-9 classification); glucose, blood counts, creatinine, and estimated glomerular filtration rate (GFR) values; and body temperature, medications, administered blood transfusion products, readmission date, and readmission diagnosis (ICD-9) if within 30 days of discharge. Non-VA hospitalization data for all subjects enrolled in Medicare will be available through a data-sharing agreement between VA and the Centers for Medicare & Medicaid Services.

## Outcomes

The clinical outcomes of potential relevance that were considered included episodes of suspected hypoglycemia and measures previously used in studies examining potential benefit of improved glycemic control such as: (1) shortened length of hospital stay; (2) fewer infections; (3) fewer episodes of acute kidney injury; (4) less need for renal dialysis; (5) lower blood transfusion requirements; and (6) less neuropathy.

LOS is selected as the primary outcome, because LOS has important cost implications, lowers the risk of hospital-acquired complications including falls and infections, and might be expected to be shortened if diabetic control can be made more efficient. It is also readily ascertainable from the EMR. Secondary outcome measures include degree of glycemic control and readmission within 30 days of discharge with the primary readmission diagnosis of control of glycemia. Tertiary outcomes include infections, acute kidney injury, and anemia, all of which have been previously used as outcome measures in studies of insulin regimens. Infection will be defined as new antibiotic administration associated with either fever or leukocytosis. Acute kidney injury is defined as a decrease in estimated GFR of greater than 50% and anemia as a drop in the hemoglobin level of at least 2 g/dL.

## Recruitment and enrollment

The POC-CT process is implemented using software tools available in CPRS. CPRS is the clinical care component of the Veterans Health Information Systems and Technology Architecture (VISTA), which supports clinical as well as administrative applications. Software tools available in CPRS include order sets (predefined customizable sets of orders), templates for clinical notes, decision logic (reminder dialog templates), and defined data objects that extract data from the medical record for display purposes (patient data objects). CPRS also has the ability to store flags (indicators in the data base) known as ‘health factors’ related to clinical parameters and flags derived from the ordering process. These tools make it possible to identify certain data elements in real time (e.g., an insulin order) and to incorporate programmatic logic into the medical record’s workflow based on the value of data elements. The order sets and templates utilized for this project were designed to be consistent in format and process with the existing system.

**Figure 1:**
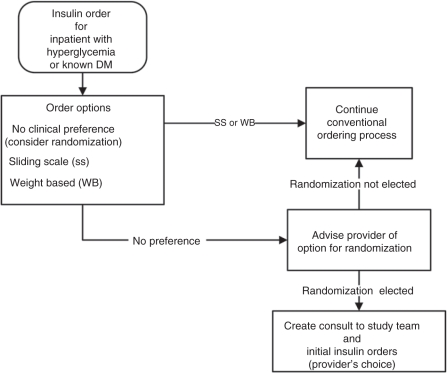
Initial order process performed by clinician

**Figure 2:**
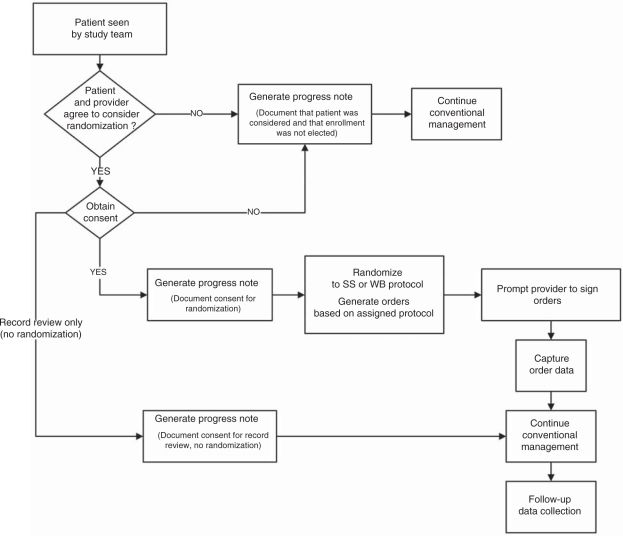
Workflow beginning when clinician has agreed to consider randomizing patient into one of two interventions

**Figure 3:**
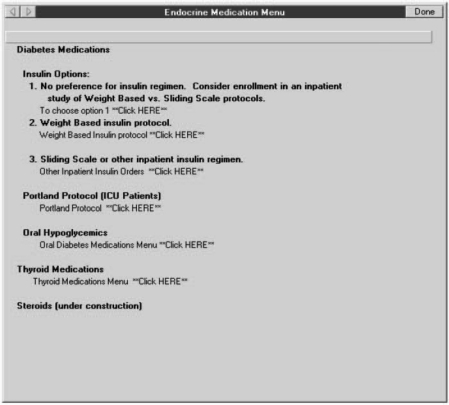
Screen shot of CPRS showing introduction of POC-CT option into the insulin options menu

The following describes the workflow of the study and demonstrates how CPRS processes already familiar to clinicians were adopted for POC-CT (Figures 1 and 2):

1. The VISTA order entry screen for insulin has been modified to include a third option in addition to the current options to order sliding scale or the weight-based regimen. The third option is labeled ‘No preference for insulin regimen, consider enrollment in an inpatient study of Weight Based vs. Sliding Scale protocols’ (Figure 3).
2. Clinicians who choose this third option will be presented with a brief description of the study and given the option to either proceed or not with consideration of their patient for study enrollment.
3. Clinicians who choose not to continue will click on the button labeled ‘No. The patient may not be approached. Proceed with usual care.’ and will be returned to the previous order entry screen to continue without further consideration of this trial.
4. Clinicians who choose to proceed will click on the button labeled ‘Yes. The research team may approach this patient for consideration of enrollment.’ and will be brought to a consult entry screen. The consult entry screen will be pre-populated requesting a ‘Research insulin dosing consent request.’ After submitting this consult, the clinician will then be directed to the order entry menu and will order either sliding scale or weight-based insulin as per their choice. This order will serve as a holding order to provide insulin treatment until the patient can be consented and randomized.
5. Upon receiving the ‘Research insulin dosing consent request,’ the study nurse will discuss the study with the patient and obtain informed consent. If the patient declines enrollment, a template progress note completing the consult will be automatically entered. Patients who refuse randomization will be asked for consent to allow access to their VISTA data for comparison to the subset of patients who accepted randomization.
6. Patients who provide consent will be randomized through the VISTA system to one of the two insulin regimens. A template progress note activated by the study nurse will document randomization. This template progress note will generate ‘health factors’ that will serve to identify patients as subjects in the trial for tracking purposes in VISTA. It will also generate the order for whichever insulin regimen the subject was randomized to receive.
7. Progress notes (for both patients accepting and declining participation) and orders (for those accepting randomization) will be automatically forwarded to the original ordering clinician.
8. By signing these documents, the clinician completes the study enrollment process.

The protocol was approved by the VA Boston Institutional Review Board (IRB) who waived Health Insurance Portability and Accountability Act (HIPAA) authorization to allow the study team, once contacted and prior to seeing the patient, to have access to protected health information in the medical record. Importantly, clinicians, in simply referring patients to the study coordinator for recruitment and signing the insulin orders generated by the randomization procedures were not considered by the IRB to be ‘engaged in clinical research’ and thus were not required to be research credentialed.

### Statistical issues

We define three main aims: (1) to determine the physician and patient acceptance of POC randomization, (2) to test the null hypothesis of no difference against reasonable alternatives (two-sided), and (3) to demonstrate successful implementation of the superior strategy. The first aim requires descriptive statistical approaches, including estimating proportions and defining patient- and physician-level predictors of acceptance. The second aim requires tuning the design parameters to achieve acceptable operating characteristics. The third aim motivates an adaptive randomization, adjusting the assignment probabilities to increase the chances that patients are assigned to the better treatment.

### Adaptive design

In the proposed study, the response or outcome is hospital LOS and the parameters of interest are the median LOS with each of the two protocols: (1) weight-based (Protocol A) and (2) sliding scale (Protocol B). We predict that the patients using the weight-based protocol will have a smaller median LOS than patients using the sliding scale protocol. To test this hypothesis, we propose using a Bayesian adaptive design.

The rules of adaptation considered herein modify the assignment probability each time the study accrues a new fixed number or ‘batch’ of patients, with practical batch sizes of at least 100 patients to allow more time for review and cleaning of data as is implicit in group sequential designs.

**Figure 4:**
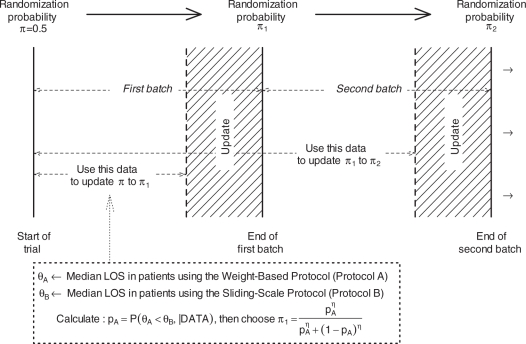
Diagram representing the flow of the design In the figure above, *π* represents the probability of assigning the weight-based protocol to a patient

According to this scheme (Figure 4)

1. First, subjects will be assigned to either weight-based protocol (Group A) with probability π = 0.5 or to sliding scale protocol (Group B) with probability 1 − π = 0.5. This assignment probability is utilized for the first batch of patients.
2. Then, the data collected on the first group of subjects are used to calculate the probability that Protocol A is superior to Protocol B given the accumulated data, that is
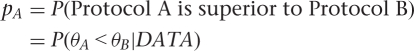

The ‘*DATA*’ here refers to the data collected on the first batch of patients, with allowance for a period (UPDATE strip in Figure 4) in which the investigators clean the data and do the update and *θ_A_* and *θ_B_* are the median LOS in Groups A and B, respectively. The ‘posterior’ probability *p_A_* (‘*probability of Protocol A being superior to Protocol B given the data*’) is calculated using Bayesian methods. Bayesian methods use prior information or beliefs, along with the current data, to guide the search for parameter estimates. Prior information/beliefs are input as a distribution, and the data then help refine that distribution and construct the posterior distribution. Our statistical model is based on an exponential data model for the LOS with conjugate Inverse Gamma prior for the median LOS [10]. Prior distributions in each group were chosen to be centered on the null median value and have a shape parameter *α*.

1. The posterior probability *p_A_* is then used to evaluate whether the accumulated information overwhelmingly supports one protocol over the other so that the termination of the trial is warranted. In particular, we would stop the trial if
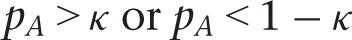

where *κ* is the *cutpoint* reflecting the level of evidence demanded by the investigators to terminate the trial. If *p_A_>κ*, then the study is terminated and Protocol A is chosen as being superior while if *p_A_<1-κ*, the study is terminated and Protocol B is chosen to be superior. The value for *κ* is at the investigators’ disposal and it is usually a value that is close to 1 (for example 0.9, 0.95, or 0.99).

1. If the decision to terminate is not made, the posterior probability *p_A_κ* is used to update the assignment probability to *π*_1_ using the transformation [11]
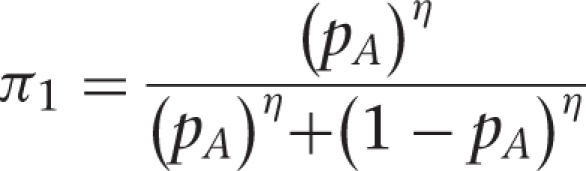

where *η* > 0 is a *calibration parameter*. If *η* is set to 1, the updated assignment probability is *π*_1_ = *p_A_*, while a value of *η* = 0 leads to a balanced randomization design. Values greater than 1 (less than 1) lead to more aggressive (less aggressive) adaptation.

1. The second batch of patients will then be assigned to Protocol A with probability *π*_1_ and to Protocol B with probability 1-*π*_1_. After the data on the second batch of patients are collected, the assignment probability *π*_1_ is updated to *π*_2_ using the above algorithm and the termination criterion is checked. If the termination criterion is met, the study is terminated. If not, the assignment probability *π*_1_ is updated to *π*_2_ using the above algorithm and the third batch is then enrolled.
2. This process is continued until either the termination criterion is met or the number of subjects enrolled reaches a pre-specified maximum number of subjects *N*_max_.

## Proposed design

Extensive computer simulations were done to select a design for the study based on their operating characteristics. The following operating characteristics were considered in selecting the final design:

1. *Overall Type I error* – the chance of declaring one of the two protocols better at any time during the trial when in fact there is no difference between the two protocols.
2. *Overall power* – the chance of declaring a protocol better at any time during the trial when in fact that protocol is better.
3. *The number of patients assigned to each protocol.* The number of patients enrolled will depend on the data collected and hence is a random variable.
4. *Time until a decision is made.* The duration of the study will depend on the data collected and hence is a random variable.

We chose a design with the following parameters: prior shape parameter *α*= 100, batch size = 200, cutpoint κ= 0.99, calibration parameter*η*= 0.5, and maximum number of patients to be randomized *N*_max_ = 3000. In addition, the updation occurs after 150 patients of each batch have entered the study, we do not update or allow stopping after the first batch, and we censor the LOS at 30 days.

We studied the above design under various scenarios. Our null hypothesis is that the median LOS with both protocols is 5 days. As alternative, we posit a minimal clinically important reduction of at least 12% in median LOS.

The operating characteristics of the design are represented in Table 1.

**Table 1:** Operating characteristics of the proposed design

Difference in median LOS (B–A) in days [median under Protocol B = 5 days]	Probability of selecting Protocol A as superior (%)	Probability of selecting Protocol B as superior (%)	Median number of patients on Protocol A	Median number of patients on Protocol B	Median duration (days)^a^
0	3	3	1495	1461	599
0.1	8	1	1634	1292	598
0.2	17	0	1738	1125	597
0.3	30	0	1791	969	595
0.4	51	0	1719	778	581
0.5	71	0	1434	598	408
0.6	86	0	1075	465	316
0.7	95	0	825	380	240
0.8	99	0	673	332	201
0.9	100	0	540	289	164
1	100	0	506	268	157

a In calculating the duration of the study, we assumed an accrual rate of 5 patients per day.

*Type I error*: Under the assumption of no difference (first row in Table 1 – median LOS is 5 days with both protocols) the probability of (incorrectly) selecting either protocol as superior was 0.06.

**Figure 5:**
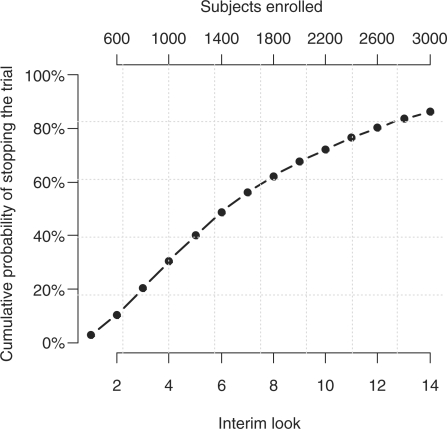
Cumulative probability of stopping the trial across interim looks; assumed median LOS with Protocols B and A are 5 and 4.4 days, respectively

*Power*: Under the alternatives (median LOS with Protocol A < median LOS under Protocol B) presented in the remaining rows of the table, the probability of correctly selecting Protocol A represents the power. For a difference of 12% in median LOS, across the interim looks, the design will correctly select Protocol A as superior with 86% probability (power), while the probability of wrongly selecting Protocol B as superior decreases fast to levels close to 0%. The decision to stop increases with time (Figure 5); thus, the probability or terminating the trial by the 6th interim look (after 1400 subjects have been enrolled) is 50% and it increases to 86% by the 14th look (after all 3000 subjects have been enrolled).

From among the many alternatives designs we evaluated, we briefly discuss here the *balanced* design that has the same parameters as the design presented above. Additional information on the simulation study including the R [12] script used in running the simulations can be obtained from the authors.

With a balanced design, the Type I error is the same, the power is slightly higher (for example, 77% *vs*. 71% to detect a difference with Protocol A of 10% in median LOS), the median number of patients enrolled is about the same (∼2000), however, while with the balanced design the enrollment is balanced, with our proposed design the number of patients assigned to the superior treatment is higher.

The operating characteristic simulation is dependent on the accuracy of the data model used to generate the LOS. In Table 1, we use the exponential model to generate the data, as well as to do the updating. Thus, it makes the assumption that the Bayesian model is correctly specified, as is done in most published work, when estimating (frequentist) operating characteristics. But the LOS data from a historical sample of patients approximating the proposed study intake criteria indicates a heavier tail, such as log-normal. Therefore, we assessed the sensitivity of the assumptions by using the log-normal model to generate the data (but still using the exponential model for the updates; Table 2).

**Table 2:** Operating characteristics under lognormal data model

Difference in median LOS (B–A) in days [median under Protocol B = 5 days]	Probability of selecting Protocol A as superior (%)	Probability of selecting Protocol B as superior (%)	Median number of patients on Protocol A	Median number of patients on Protocol B	Median duration (days)^a^
0	4	3	1469	1473	599
0.1	8	2	1594	1317	599
0.2	16	1	1711	1163	597
0.3	28	0	1759	998	595
0.4	46	0	1724	832	587
0.5	62	0	1600	696	485
0.6	78	0	1244	535	360
0.7	90	0	924	414	275
0.8	96	0	715	352	210
0.9	99	0	626	309	193
1	100	0	522	278	160

a In calculating the duration of the study, we assumed an accrual rate of 5 patients per day.

The difference between these two simulations illustrates the modest sensitivity of the operating characteristics to misspecification of the data model. For example, the Type I error estimate rises from 6% to 7%, and the power at a difference of 0.5 days drops from 71% to 62%. However, we consider the Type I error less relevant in this context, comparing the effectiveness of two widely used procedures for setting dose. In a different context, the Type I error might be more important. The probability of making the right choice when it matters (a full day difference) is high (100%) in the log-normal scenario, too. These results illustrate the value of a hybrid approach, where the Bayes method is confined to updating the randomization probability (thus closing the implementation gap and maximizing the number of patients receiving the right treatment) and inference is based on operating characteristics from a range of more realistic models.

## Discussion

POC-CT methodology is well suited for studies with the following features:

- Interventions already approved by the FDA.
- A clinical question where there is equipoise regarding clinically relevant alternative interventions.
- Interventions that are part of routine practice, well tolerated, and have well-recognized toxicities which mitigates the need for adverse event monitoring beyond that in routine clinical care.
- Subject identification, inclusion and exclusion criteria, and endpoints that are accurately obtained from the EMR.
- Outcomes are objective and require little or no adjudication.
- Study protocol requiring minimal deviations from usual care.
- No systematic laboratory or clinical follow-up required for either safety or comparative effectiveness.

This trial is designed to be on the pragmatic extreme of the clinical trial spectrum with the subject consent process being the sole perturbation of the clinical care ‘ecosystem.’ The absence of study specific interventions, procedures, and monitoring together with passive data capture attempts to maximize the relevance of the findings to current practice at the VA Boston Healthcare System. Adaptive randomization is designed to assign subjects preferentially to the treatment arm that, in real time, appears superior, with an ‘efficacy’ stopping rule that has acceptable Type I error. If the study terminates without reaching its ‘efficacy’ boundary, it will reliably rule out a substantial difference, in which case cost, convenience, and other factors will dictate which treatment arms continue to be supported. Such direct translation of study results into clinical practice defines a ‘learning healthcare system.’

The clinical question posed in this protocol, comparison of insulin administration methods, was chosen because it is amenable to a maximally pragmatic study as defined by the PRECIS criteria and because:

- Broad participation by healthcare providers is expected. The clinical question is compelling and in practice there is apparent equipoise between the two regimens in that roughly half of patients are currently treated by each technique.
- The inclusion/exclusion criteria will allow enrollment of nearly all the VA Boston patients who require the intervention.
- The study interventions are currently utilized at VA Boston, have known toxicities that are monitored as part of usual care, and thus require no specific study related monitoring.
- All study data elements are objective, resident in the EMR and do not require study specific interactions or visits for capture.
- Adaptive randomization methodology leads to real-time incorporation of study results into practice, if one treatment proves superior.

The ability to implement this study is made possible by the VA’s EMR environment. CPRS is in use at all the VA’s 1500-plus points of care and was designed to incorporate clinical data as part of efforts to improve clinical care. As a result, it features several packages that allow end users to automatically generate reports, ‘listen’ for certain values associated with patient data objects, consider these values with programmatic logic, and introduce information and workflows directly into the EMR. To capitalize on this level of flexibility, most VA healthcare systems employ Clinical Application Coordinators, who use these tools to create and report measures of the quality of care, to implement guidelines, and to create clinical reminders based on the priorities of each hospital. This infrastructure will allow for the relatively easy roll-out of this and other POC-CT studies system-wide as well as systematic implementation of findings.

The ability to use existing functionalities, as opposed to developing custom software is important for a number of reasons. First, development of new software functionality is constrained by time for development, testing, and approval, and development resources. Second, by capitalizing on existing system functionality, we increase the likelihood of a successful deployment to other VA hospitals or clinics, each one of which employs CPRS. Finally, although this particular use of CPRS may be novel, the POC-CT processes are presented through familiar interfaces and into a culture of robust CPRS use, which we hope will facilitate adoption of this approach.

The ability of institutions to implement POC-CTs is dependent on the ability to use the EMR to: (1) identify events as they present in real time; (2) intervene in the clinical care workflow; and (3) track longitudinal data. It is worth noting that these functionalities are critical to the creation and implementation of many novel approaches to learn from and improve healthcare based on real data and that few systems offer such capabilities to end users. The need for such functionalities is of particular relevance in light of the US Federal Government’s upcoming investment of $19 billion to support the adoption of EMRs [13]. Much of this funding is contingent on the adoption of ‘certified’ EMR systems and the ‘meaningful use’ of such systems. Definitions that require flexible integration with EMR data and workflows are essential to meeting the goals of such enormous investments [14].

The ethical and practical considerations of informed consent have been extensively discussed and debated [15–19] as have methods such as cluster randomization which might obviate or preclude individual informed consent [20,21]. Detailed analyses of these considerations are outside the scope of this article. However, as POC-CTs or similarly designed trials become an important component of clinical research, it will be incumbent on investigators, ethicists, and IRBs to fully consider the potential benefits and apparently minimal incremental risks of a POC-CT, and to take responsibility for helping their healthcare systems to lower the barriers to successful study design and implementation of improvements in care.

A study coordinator will obtain written informed consent for all subjects entered into this trial. This requirement accounts for a significant proportion of the study cost and introduces the single most tangible perturbation to the usual care workflow. We recognize that replacement of such full written informed consent by an alternative (such as simple ‘notification’ by the healthcare provider and verbal consent by the subject with subsequent randomization through a fully automated computerized process) would result in an even more efficient design, with a closer match to clinical care. The IRB could consider such a variation on the usual research informed consent, on a study-by-study basis, especially when the POC-CT results in care materially identical to usual clinical practice. Parallel requirements would be a waiver of HIPAA authorization to obtain study data from the EMR and acknowledgement that treating clinicians who authorize automated randomization are not ‘engaged’ in research.

A POC-CT will likely require significantly less study-specific infrastructure and cost than traditional RCTs (after the up-front investment in coordinating center and informatics, already made by the VA). These advantages together with an economy of scale once an investment in the methodology has been made could lead to low incremental cost per study as well as allowing study designs of sufficient duration to capture clinically relevant (as opposed to surrogate) endpoints.

## Limitations

Several issues may impede adoption of POC-CTs. Some patients may find it surprising and troubling that healthcare providers do not know what is the best treatment for them. This disclosure could make the consent process lengthy and difficult. Although the medical community might be at equipoise regarding treatment options, individual healthcare providers may have strong treatment preferences, either in general or for particular individual patients. Both of these issues could have ramifications for recruitment rates and the success of a POC-CT. We note that ‘reluctance to randomize’ is an issue for all RCT designs, not just POC-CT.

Most (if not all) uses of POC-CT we envision would have an open (unblinded) design, which raises the possibility of cross-contamination of treatments, or differential clinical interventions due to physicians’ perceptions of patients’ needs, or other failures of the exclusion principle, such as observational bias in the outcome. Therefore, the use of POC-CT may be restricted to clinical situations where the effects are likely to be minimal. We think that the EMR-based protocols we compare here, as well as the outcome of LOS, sharply reduce physician unblinding as a threat. We emphasize that POC-CT is not a universal alternative to the classical double-blind RCT with its many controls for bias; rather, it can be seen as a competitor to observational studies, by removing the particular bias from selection by indication that plagues such non-experimental studies.

Our pragmatic intent requires us to rely on individual clinician judgment of eligibility, which is another mark of distinction between POC-CT and conventional trials, which often have elaborate procedures for defining ‘inclusion and exclusion.’ This certainly restricts the use of POC-CT to contexts where such precision is unnecessary. However, it also contributes to the ‘ecological validity’ of treatment effects.

Highly pragmatic POC-CTs such as this study may yield results that are locally convincing but are not easily generalized to other healthcare systems. A healthcare system such as the VA, motivated to conduct POC-CTs and with the organization and infrastructure capable of supporting it, could generate ‘locally selfish’ evidence-based medicine to gain evidence of comparative effectiveness most relevant to its population and systems. In general, comparative effectiveness findings are most applicable to the systems and individuals who participated in its creation rather than to the ‘free riders’ – those who may desire evidence-based medicine but who are unwilling to be a part of that evidence.

The above may suggest that the POC-CT approach is limited to a narrow range of clinical questions and contexts. We are just now beginning to expand our list of possible use cases, and we do not want to speculate in advance of the facts. We agree with Vickers and Scardino [9] that features of POC-CT might be implemented in practice in four distinct areas: surgery, ‘me too’ drugs, rare diseases, and lifestyle interventions. In addition to questions of optimizing care (such as the insulin example described here) use cases currently under consideration include technology introduction (imaging, robotics, and biomarker-guided therapy), pre-hydration with bicarbonate *versus* saline with or without n-acetylcysteine in contrast-induced nephropathy, and comparing prolonged exposure and cognitive processing therapies as alternative treatment strategies for post-traumatic stress disorder.

Finally, the proposed study design using outcome adaptive randomization leads to real-time implementation into practice, and stimulates reconsideration of the role of the traditional peer review process that subjects study results to expert outside review before planning their implementation in practice.
